# *Lactococcus lactis* provides an efficient platform for production of disulfide-rich recombinant proteins from *Plasmodium falciparum*

**DOI:** 10.1186/s12934-018-0902-2

**Published:** 2018-04-05

**Authors:** Susheel K. Singh, Régis Wendpayangde Tiendrebeogo, Bishwanath Kumar Chourasia, Ikhlaq Hussain Kana, Subhash Singh, Michael Theisen

**Affiliations:** 1 Department for Congenital Disorders, Statens Serum Institut, Artillerivej 5, 2300 Copenhagen S, Denmark; 2 Centre for Medical Parasitology at Department of International Health, Immunology and Microbiology, University of Copenhagen, Copenhagen, Denmark; 3 Department of Infectious Diseases, Copenhagen University Hospital, Rigshospitalet, Copenhagen, Denmark; 4 Indian Institute of Integrative Medicine, Jammu, India

**Keywords:** *Plasmodium falciparum*, Malaria, Disulfide-rich protein, Merozoite antigens, *Lactococcus lactis*

## Abstract

**Background:**

The production of recombinant proteins with proper conformation, appropriate post-translational modifications in an easily scalable and cost-effective system is challenging. *Lactococcus lactis* has recently been identified as an efficient Gram positive cell factory for the production of recombinant protein. We and others have used this expression host for the production of selected malaria vaccine candidates. The safety of this production system has been confirmed in multiple clinical trials. Here we have explored *L. lactis* cell factories for the production of 31 representative *Plasmodium falciparum* antigens with varying sizes (ranging from 9 to 90 kDa) and varying degree of predicted structural complexities including eleven antigens with multiple predicted structural disulfide bonds, those which are considered difficult-to-produce proteins.

**Results:**

Of the 31 recombinant constructs attempted in the *L. lactis* expression system, the initial expression efficiency was 55% with 17 out of 31 recombinant gene constructs producing high levels of secreted recombinant protein. The majority of the constructs which failed to produce a recombinant protein were found to consist of multiple intra-molecular disulfide-bonds. We found that these disulfide-rich constructs could be produced in high yields when genetically fused to an intrinsically disorder protein domain (GLURP-R0). By exploiting the distinct biophysical and structural properties of the intrinsically disordered protein region we developed a simple heat-based strategy for fast purification of the disulfide-rich protein domains in yields ranging from 1 to 40 mg/l.

**Conclusions:**

A novel procedure for the production and purification of disulfide-rich recombinant proteins in *L. lactis* is described.

**Electronic supplementary material:**

The online version of this article (10.1186/s12934-018-0902-2) contains supplementary material, which is available to authorized users.

## Background

Protein production is often the limiting factor in research and development of vaccines, sero-diagnostic tools, and immuno-assays. The yield, purity and cost of production are some of the important parameters which determine the choice of expression system for recombinant protein production. There is no single system, which can accommodate all recombinant proteins, and if successful, expression yields vary by orders of magnitudes due to biophysical and structural properties of the recombinant proteins. Of the different proteins, the production of homogenously folded disulfide bonded proteins have been particularly challenging [1, 2]. The lactic acid bacteria *Lactococcus lactis* has gained importance as a host for heterologous protein expression [3–7] because (1) it can export the recombinant protein into the culture supernatant from where it can be readily purified, (2) it is a Gram positive bacteria which does not produce endotoxins, (3) unwanted glycosylation of proteins has not been described, and (4) it is ‘generally recognized as safe’ (GRAS). The *L. lactis* expression system is compatible with large-scale upstream and downstream processes and therefore provides a low cost production system. The safety of this expression system has been demonstrated in humans (reviewed in [8, 9]). Recently, we have reported use of *L. lactis* for successful production of recombinant *Pf*s48/45, a disulfide-rich malaria transmission blocking vaccine candidate from *Plasmodium falciparum*, which has been difficult to produce as recombinant protein with correct conformation using a variety of other prokaryotic as well as eukaryotic recombinant protein expression systems.

In this study we have further demonstrated the suitability of *L. lactis* for expression and purification of a panel of 31 recombinant proteins from 25 distinct antigens of *P. falciparum* (non-overlapping sub-domains from some antigens were expressed separately to study their distinct antigenic properties) as several of which consist multiple structural disulfide bonds. Proper disulfide bond formation of the recombinant antigens is essential for not only the native structure and its antigenic property but also for inducing appropriate innate and adaptive immune responses. The antigens selected in this study are localized in diverse sub-cellular compartments of the merozoite, the transient extracellular stage of the asexual blood stage of *P. falciparum*, which is highly specialized for erythrocyte invasion [10, 11]. These include the integral merozoite membrane proteins, the associated peripheral surface proteins and proteins secreted from the various secretory organelles such as the micronemes, rophtries and the dense granules which are understood to form functional complexes with merozoite surface proteins [12]. Together these merozoite surface protein complexes play important roles in erythrocyte invasion and several of them have been identified as putative targets of protective immunity against malaria [13]. The panel of malaria antigens selected in this present study covers a variety of protein structural complexities, ranging from proteins predicted to have a highly disordered structure to those consisting of multiple (2–75) cysteine residues predicted to form structural disulfide bonds determining the overall secondary and tertiary structure of the respective proteins. The panel of malaria antigens consists of merozoite antigens which are targets of (1) naturally acquired immunity, (2) functional antibodies, and (3) include both variable and conserved regions of the respective antigens. We show that *L. lactis* is an efficient system for expression of recombinant malaria antigens with a variety of protein structural complexities.

## Results

### Protein expression

**Fig. 1:**
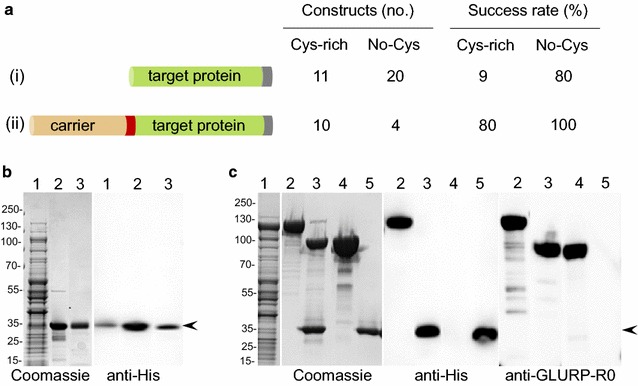
Expression and Purification of target recombinant proteins in *L. lactis*. **a** Left panel: Schematic representation for cloning and expression of target recombinant proteins from *P. falciparum* antigens without (i) and with (ii) carrier fusion protein; Right panel: Classification of the target recombinant proteins based on their cysteine content (constructs containing two or more cysteine residues are classified as cysteine-rich proteins) and the overall success rates for production of target recombinant proteins by the respective *L. lactis* expression clones using strategy (i) or (ii). **b** Expression and purification of recombinant cMSP3^3D7^ (a representative recombinant protein which does not contain cysteine residues and expressed successfully in *L. lactis* without carrier fusion protein); Left panel: Coomassie blue-stained non-reducing SDS-PAGE analysis showing purification of recombinant cMSP3^3D7^protein secreted in *L. lactis* culture supernatant; Lane 1: raw culture supernatant, lane 2: Ni^+^-NTA purified cMSP3^3D7^; lane 3: Ion-exchange purified cMSP3^3D7^; *Right panel*: Western Blot analysis of recombinant cMSP3^3D7^ using anti-His antibody. **c** Expression and purification of recombinant R0-MSPDBL2 (a representative cysteine-rich recombinant protein and which was expressed successfully in *L. lactis* with GLURP-R0 as a carrier fusion protein); Left panel: Coomassie blue-stained non-reducing SDS-PAGE analysis showing purification of recombinant R0-MSPDBL2 protein secreted in *L. lactis* culture supernatant; Middle panel: Western blot analysis of recombinant R0-MSPDBL2 using anti-His antibody; and Right panel: Western blot analysis of recombinant R0-MSPDBL2 using anti-GLURP-R0 antibody; Lane 1: raw culture supernatant; lane 2: Affinity purified R0-MSPDBL2 recombinant protein; lane 3: TEV cleavage of recombinant R0-MSPDBL2 and SDS-PAGE separation of R0 and MSPDBL2 fragments; Lane 4: Ion-exchange chromatography separates cleaved R0 carrier protein after TEV protease cleavage and Lane 5: Purified recombinant MSPDBL2 obtained after TEV cleavage and ion-exchange chromatography. The arrow-heads indicate positions of cMSP3^3D7^ and MSPDBL2 recombinant proteins in respective panels. Numbers shown on the left hand side of the gel pictures represent positions of protein molecular weight markers in kDa

A panel of *P. falciparum* proteins targeted by naturally acquired immune responses was produced as C-terminally His-tagged proteins in *L. lactis* MG1363 (Fig. 1a(i)). Sub-domains of some malaria antigens representing the variable and conserved regions (such as from MSP2 to MSP3) were expressed as separate recombinant proteins to allow detection of both allele-specific and cross-reactive antibodies (Table 1). The success of recombinant protein production from the respective *L. lactis* clone was determined (as Low, Medium or High) as per the yield of the secreted recombinant protein purified from a batch fermentation of 1 l. We observed an overall efficiency of 55% with 17 out of 31 recombinant clones producing a secreted recombinant protein as detected by SDS-PAGE and Western Blot analysis (Table 1). The success rate was much higher (80%) for recombinant proteins which lacked cysteine residues (expression was detected for 16 out of 20 such recombinant proteins). However, the success rate was lower (9%) for those recombinant proteins containing two or more cysteine residues (expression was detected for only 1 out of 11 recombinant proteins). In general, expression constructs which produced high yields of recombinant proteins were low in cysteine residues and relatively unstructured (Additional file 1a). In contrast, neither the iso-electric point (*p*I) nor the molecular weight of the cloned expression construct seemed to affect its expression level in *L. lactis* (Additional file 1b). We therefore speculated that increasing the overall disorder score of the difficult-to-express proteins by creating a fusion protein with an unstructured protein domain might enhance the yield of the desired recombinant protein. Accordingly, constructs which failed to produce a recombinant protein were genetically linked to an unstructured carrier protein (the R0 sub-domain of the GLURP antigen) (Fig. 1a(ii)). A TEV protease cleavage site was inserted in the fusion-junction to allow removal of the R0 carrier protein. Twelve out of fourteen fusion recombinant constructs thus designed, showed higher expression levels compared to the respective target proteins expressed without fusion with the disordered carrier protein (Fig. 1a). Two constructs with large number of cysteine residues and highly complex predicted secondary structure (EBA140RII and RIPR containing 23 and 75 cysteine residues, respectively) did not show improvement in their expression levels despite their fusion with the carrier protein.

**Table 1:** Characteristics and purification strategies of recombinant proteins

Antigen^a^	Accession number	Target sequence	Cysteine (no)	Disorder tendency^b^	Expression level		Capturing		Polishing		Mass (Da)	
					No carrier	With carrier	IMAC	Heat	IEC	Yield (mg/l)^c^	Calculated	Observed^d^
GPI-anchored												
MSP1_19k_	PFI1475W	N1607–N1702	12	0	High		X		A	30	12.2	15
MSP2^3D7^	PFB0300C	A111–G238	0	100	High		X		A	20	13.7	40
MSP2^FC27^		E143–G230	0	94	High		X		A	20	9.6	25
Pf12	PFF0615C	K155–S323	6	0	Low	Med		X	C	10	20	20
Pf38	PFE0395C	K139–I327	6	0	Low	Med		X	C	10	23	25
Peripherally associated												
nMSP3^3D7^	PF10_0345	N21–L182	1	25	High		X		A	40	17.6	27
cMSP3^3D7^	PF10_0345	K183–H354	0	0	High		X		A	18	20	30
nMSP3^K1^	PFU08851	N21–L208	0	48	High		X		A	35	21	37
MSP6	PF10_346	Y17–N160	0	70	Med		X		A	10	17.5	23
MSP3.3	PF10_0347	Q23–T227	0	48	Med		X		A	7	22.3	37
MSP3.7	PF10_0352	K27–R213	0	100	Med		X		A	7	20.9	37
GLURP-R0	AAA50613	T27–A500	0	99	High		X		A	75	54.3	90
GLURP-R2	AAA50613	V705–1177	0	78	High		X		A	35	53	110
SERA5	PF02_0072	K17–192/226–S382	8	40	Low	High		X	C	17	35.5	38
Pf41	PFD0240C	S231–S378	6	0	Low	Med		X	C	7	18	18
MSPDBL1	PF10_0348	K143–D443	13	0	Low	Med		X	C	8	37	37
MSPDBL2	PF10_0355	K161–L457	12	0	Low	High		X	C	15	36.5	35
MSPDBL1 (Leucine)	PF10_0348	D508–K697	0	69	Med		X		A	7	22	35
Micronemal/microneme												
EBA140RIII–V	MAL13P1.60	E746–T1045	0	93	High		X		A	16	32.9	40
EBA140 RII	MAL13P1.60	Q146–K710	23	0	Low	Low		X	C	1,5	67.3	85
GAMA	PF08_0008	N25–H337	7	10	Low	Med		X	A	5	36.8	45
RIPR	PFC1045C	K279–D995	75	0	Low	Low		X	A	1	82.3	110
SUB2	PF11_0381	K14–N681	2	29	Low	Med		X	A	5	78.3	90
Rhoptry												
PfRh2b	MAL13P1.176	L2790–K3184	0	91	Med		X		A	8	44.7	120
PfRh2a	PF13_0198	H2874–S3060	0	26	High		X		A	13	22	25
PfRh4.2	PFD1150C	L1275–I1451	0	11	Low	Med	X		C	2	21.5	21
PfRh2-2030	PF13_0198	E2030–Q2528	1	0	Med		X		A	8	58	58
RAMA	MAL7P1.208	I42–N786	0	100	Low	Med	X		A	7	90.6	120
RALP-1	MAL7P1.119	N396–P634	0	47	Low	Med	X		A	10	27	37
RON2	PF14_0495	I775–K958	0	49	Med		X		A	10	24	25
RON4	PF11_0168	K25–L709	0	100	Low	Med	X		A	9	76	150

*A* anion exchange (HiTrap Q HP); *C* cation exchange (Hitrap SP HP)

^a^Gene originate from 3D7 unless otherwise stated

^b^Disorder prediction score was calculated by using IUPred [41]

^c^The levels of expressed protein was determined by BCA kit after final step purification. Expression levels are grouped into “low” (0.1–2 mg/l), “medium” (2–10 mg/l), and “high” (> 10 mg/l)

^d^By SDS-PAGE

### Protein purification

A simple workflow was developed consisting of batch-fermentation and a 2-step downstream purification process. Recombinant proteins from *L. lactis* expressing clones were secreted in the culture medium and harvested by separation of cellular biomass through centrifugation and affinity purified through the His-tag using HisTrap HP-column. Upon elution the fractions containing the recombinant protein were collectively subjected to a polishing step using ion-exchange chromatography to further remove contaminating host-cell-protein (HCP). Using this approach a highly purified recombinant protein was obtained as exemplified with recombinant cMSP3^3D7^ (Fig. 1b). Recombinant proteins expressed with R0-fusion partner were harvested from the clarified culture medium using the same procedure as described above and exemplified with recombinant R0-MSPDBL2 (Fig. 1c). The affinity purified fusion protein (Fig. 1c, lane 2) was cleaved with the TEV protease (Fig. 1c, lane 3) before being purified further with ion-exchange chromatography column (Table 1) to separate HCP, TEV, and the R0-fusion partner (Fig. 1c, lane 4) from the target recombinant protein (Fig. 1c, lane 5). The identity and purity of the target protein was confirmed at each step by immune blotting with antibodies against the His-tag and R0 to demonstrate removal of the carrier protein (Fig. 1b, c).

### GLURP stabilizes structural domains against thermal denaturation

**Fig. 2:**
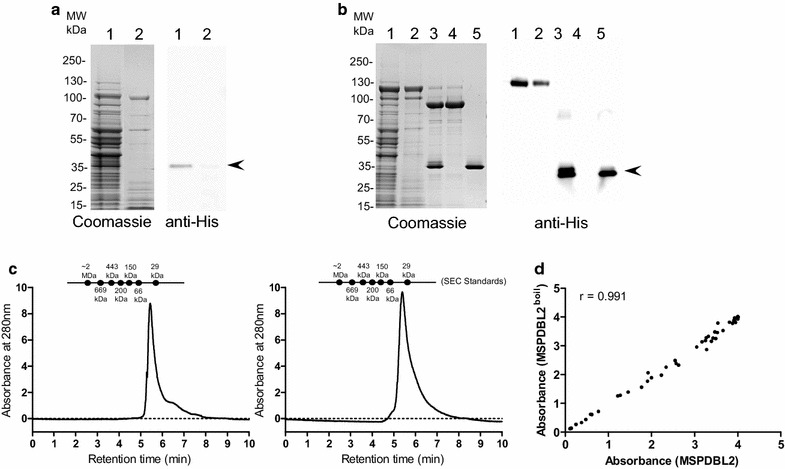
Purification of target recombinant protein by heat-treatment. **a** Heat treatment of *L. lactis* raw culture supernatant expressing recombinant MSPDBL2; Left panel: Coomassie blue stained SDS-PAGE analysis of MSPDBL2 containing supernatant before (lane 1) and after (lane 2) heat treatment; Right panel: Corresponding Western blot probed with anti-His antibodies. **b** Heat treatment of *L. lactis* raw culture supernatant expressing R0-MSPDBL2 fusion protein; Left panel: Coomassie blue stained SDS-PAGE analysis of R0-MSPDBL2 containing culture supernatant before (lane 1) and after (lane 2) heat treatment; lane 3: TEV protease treatment of recombinant R0-MSPDBL2 and ion-exchange chromatography based separation of R0 fusion partner (lane 4) and MSPDBL2 (lane 5) fragments after TEV protease cleavage of R0-MSPDBL2 derived from heat treated culture supernatant; Right panel: Corresponding Western blot probed with anti-His antibodies. The arrow-heads indicate the position of MSPDBL2 fragment. **c** Size exclusion chromatography analysis of purified recombinant MSPDBL2 as obtained from *L. lactis* culture supernatants expressing R0-MSPDBL2 (left panel) and MSPDBL2 expressed without carrier fusion partner (right panel). SE-HPLC was performed under native conditions in a phosphate buffer pH 7.2 to determine the amount of monomer in the sample. **d** Comparative antigenicity assessment of purified recombinant MSPDBL2 protein. The scatter-plot shows Pearson’s co-relation coefficient between the ELISA values observed for reactivity of a panel of 54 malaria hyperimmune Liberian sera samples against the recombinant MSPDBL2 protein preparations purified by heat treatment (y-axis) or without heat (x-axis)

The GLURP-R0 region is highly disordered as predicted by the IUPred software and is characterized to have low hydrophobicity and high net charge (Table 1). It migrates on SDS-PAGE with an apparent molecular mass more than twofold higher than its predicted molecular mass. Disordered proteins are often heat-resistant [14] and since boiling has been used as a strategy to purify similarly disordered recombinant epsin 1 and AP180 [15], we speculated that GLURP-R0 might stabilize its fusion partners against thermal denaturation. In a first experiment, we cultured the MSPDBL2 construct without (Fig. 2a) and with (Fig. 2b) a R0-fusion partner. Boiling of culture supernatants from the batch fermentation of recombinant clones expressing R0-fusion constructs and its immediate cooling in an ice-bath resulted in the denaturation of > 80% of the HCP, which was subsequently removed by centrifugation (Fig. 2a, b, lane 2). The MSPDBL2 recombinant protein did not resist heat-treatment (Fig. 2a, lane 2) whereas the R0-MSPDBL2 fusion protein remained in solution under these conditions (Fig. 2b, lane 2). The supernatant containing R0-MSPDBL2 was incubated with the TEV protease (Fig. 2b, lane 3) and the resulting mixture was subjected to purification through ion-exchange chromatography column to separate TEV-protease, R0-fusion partner and HCP (Fig. 2b, lane 4) from the target protein (Fig. 2b, lane 5). The integrity of the resulting MSPDBL2-domain was examined by SE-HPLC and ELISA. For comparison, recombinant MSPDBL2 purified without heat-treatment was included in the analysis. The two MSPDBL2 recombinant proteins migrated with similar retention times of 5.54 min by SE-HPLC (Fig. 2c) suggesting that heat treatment did not affect protein-stability and multimerization. The two MSPDBL2 protein preparations also showed similar antigenicity profiles against sera from malaria-immune individuals suggesting that the overall protein structure of the cysteine-rich DBL2 domain is unaffected by the heat-treatment (Fig. 2d). Next, we applied the same heat-treatment and purification procedure to the remaining nine R0-fusion proteins (Table 1).

### Comparative assessment of purified recombinant merozoite proteins

**Fig. 3:**
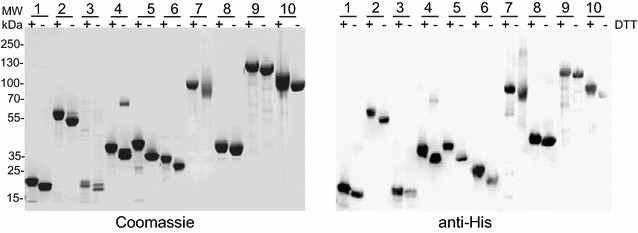
Characterization of cysteine-rich recombinant proteins purified by heat treatment method. Left panel: Coomassie blue-stained SDS-PAGE analysis of purified recombinant proteins under non-reducing or under reducing (upon treatment with DTT) conditions. Lanes 1: pf12; 2: SERA5; 3: Pf41; 4: MSPDBL1; 5: MSPDBL2; 6: Pf38; 7: EBA140 RII; 8: GAMA; 9: RIPR and 10: SUB2. Right panel: Corresponding Western Blot analysis using anti-His antibody

Most of the purified cysteine-rich recombinant proteins migrated as a single band by SDS-PAGE under non-reducing conditions with apparent molecular weights, which are in agreement with those predicted from the deduced amino acid sequence (Fig. 3). The addition of a reducing agent resulted in a band shift in the mobility of these recombinant proteins on SDS-PAGE demonstrating that these recombinant proteins consist of disulfide-bonds. Disulfide-bonding in these protein preparations was further confirmed by demonstrating no or very low levels (< 1%) of free thiol under native condition. SDS-PAGE analysis also showed that many of the non-disulfide bonded proteins and particularly those with high predicted disorder scores migrated with much higher apparent molecular weights than their respective predicted MWs (Additional file 2). As expected, a strong correlation between anomalous migration as observed by SDS-PAGE and disorder score (Additional file 3, r 0.7554) was observed.

**Fig. 4:**
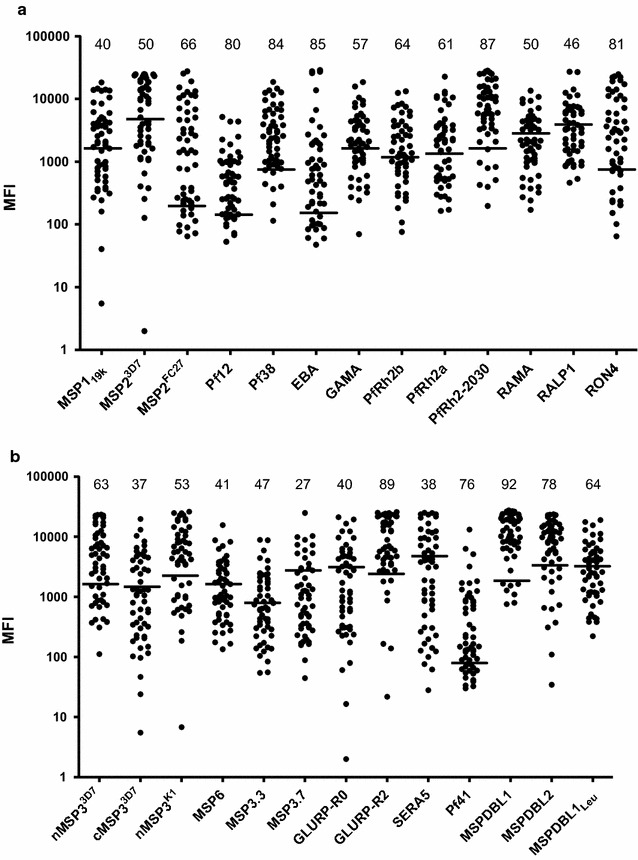
Antigenic analysis of different target recombinant proteins expressed and purified from *L. lactis* culture. Total IgG prevalence as observed in 54 malaria hyperimmune Liberian sera against purified recombinant proteins as determined through multiplex analysis. **a** Antigenic analysis of recombinant proteins derived from membrane anchored *P. falciparum* antigens which include the GPI-anchored and transmembrane domain containing antigens from secretory organelles (micronemes and rhoptries). **b** Antigenic analysis of recombinant proteins derived from *P. falciparum* antigens which are peripherally associated with merozoite surface. The horizontal lines mark the cutoff-levels determined by the mean fluorescence intensity (MFI) +2 standard deviations of reactivity of sera from Danish donors who have never been exposed to malaria infection. The numbers listed above scatter plot for each antigen represent the percentage of hyperimmune sera which show positive reactivity against the respective antigen

The overall structure of the purified recombinant proteins was also examined by assessing their antigenicity in a multiplex assay (MPA). All antigens were recognized by naturally acquired antibodies in plasma from hyper immune Liberian blood donors (Fig. 4).

## Discussion

Lactic acid bacteria have a long history of use in the production of fermented foods. More recently, they have also gained importance as microbial cell factories for the production of pharmaceuticals [8, 9]. Development of new vaccines and sero-diagnostic tools constantly pose challenges to the identification of convenient and potent expression systems for recombinant protein production. Here, we have shown that the *L. lactis* expression systems is ideal for the production of malaria antigens because it (i) accommodates cysteine-rich proteins (ii) offers a scalable fermentation process (iii) allows secretion of the recombinant protein into the culture medium thereby simplifying the purification process, and (iv) exhibits similar codon bias as *P. falciparum* and therefore does not require codon optimization prior to protein expression.

*Plasmodium falciparum* merozoite antigens have previously been produced in *E. coli* [16], wheat germ cell–free expression system [17] and HEK293 cells [18]. Whereas each of these systems proved successful for some antigens, they also present distinct challenges with respect to producing correct post-translational modifications which usually determine the quality and activity of the target recombinant protein. In eukaryotes such as *P. falciparum*, the oxidizing environment of the endoplasmic reticulum (ER) provides a milieu for disulfide bond formation [19]. Eukaryotic expression systems may potentially form the native disulfide bonds. In contrast, prokaryotic organisms lack the sophisticated ER machinery of and show considerable diversity in their mechanisms and capacity for formation of protein disulfide bonds [20]. Amongst prokaryotes, *E. coli* has long been the first choice for recombinant protein production potentially giving very high yields. However, expression of disulfide-bonded proteins in *E. coli* remains challenging and use of different *E. coli* strains have been proposed to overcome some of these difficulties [2]. Though the mechanisms which control the formation of disulfide bonds in *L. lactis* are uncharacterized, this system has proved an efficient host for the production of disulfide bonded fragments of *Pf*s48/45 [21–25].

Glycosylation is another common protein post-translational modification likely to affect the antigenic properties of the recombinant protein depending on the host cell system used for its expression [26]. Since, most of the *P. falciparum* antigens are non-glycosylated, using eukaryotic expression systems which perform host-specific glycosylation, may compromise essential epitopes of these antigens. To avoid glycosylation these eukaryotic systems necessitate mutation of N-linked glycosylation sites, which in turn might alter functionality [27] and possibly antigenicity and immunogenicity of the recombinant protein. In contrast, *L. lactis* does not perform unwanted glycosylation and has proven useful for the production of a disulfide-bonded malaria protein [21]. In general, we feel that *L. lactis* has following advantages over *E. coli* for expression of malaria recombinant proteins: (1) Codon-optimization of the recombinant malaria gene is not required for obtaining successful expression in *L. lactis*; (2) Recombinant protein is secreted in the *L. lactis* culture supernatant making the down-stream processing much easier and less-expensive; and (3) There is no lipo-polysaccharide contamination in *L. lactis* expression.

In the present study, we have produced a panel of merozoite antigens consisting of proteins with and without disulfide bonds. We found an overall success rate of 55% but it was also evident that the success rate for disulfide-bonded proteins was lower than that for cysteine-poor proteins (9% vs. 80%). In general, the production yield of relatively disordered protein fragments was higher than that of ordered protein regions. The exact reason for this is unknown but it may be speculated that higher solubility, increased stability, or a more efficient transportation across the cell membrane may play a role. Though the underlying mechanism is not clear, addition of an intrinsically disordered protein fragment increased the overall yield of these difficult-to-express protein fragments. Eight out of 10 disulfide bonded proteins produced high levels of recombinant protein when fused to the GLURP R0-region.

GLURP belongs to a group of proteins lacking ordered structure [28, 29], known as intrinsically disordered proteins (IDP). These proteins exist without a well-defined folded structure, having a highly biased amino acid composition with no cysteine and aromatic residues, an overall low hydrophobicity level, and an extremely high content of charged residues (Reviewed in [14, 30, 31]). The relatively, low content of hydrophobic amino acid residues may explain the anomalous migration by SDS-PAGE observed in our study. A deficiency in hydrophobic amino acid residues may result in less binding of SDS [32, 33], thereby slowing migration and therefore resulting in an apparent overestimation of the molecular mass of the recombinant protein through SDS-PAGE analysis.

In the present study we argue that these peculiarities of the amino acid sequences of the intrinsically disorder protein domain of the fusion proteins increased the overall protein expression efficiencies by increasing solubility, resistance to aggregation and possibly by facilitating the translocation of otherwise tightly folded proteins across the cell membrane. The lack of a stable globular structure provides IDP with several extraordinary features including the ability to be unharmed by prolonged heat treatment ([31] and references therein). We have for the first time exploited this feature to provide a highly structured protein with the same indifference to heat treatment. This ability of the IDP was first explored by creating a protein fusion with the disulfide-bonded MSPDBL2. Our results clearly demonstrate that GLURP-R0 protects the integrity of the MSPDBL2 domain from boiling. The MSPDBL2 domain purified by heat-treatment and by conventional purified showed similar HPLC and antigenicity profiles. In contrast, more than 80% of the HCP was eliminated by this heat treatment step possibly because of a low content of intrinsically disordered proteins in *L. lactis* culture supernatants. The applicability of this method was further examined by creating protein fusions between GLURP-R0 and nine other disulfide-rich merozoite antigens. All fusion proteins were heat stable and the resulting disulfide-rich merozoite antigens were strongly recognized by naturally acquired *P. falciparum* antibodies with levels and prevalence of specific IgG antibodies which were similar to those observed with malaria antigens produced in several other systems [17, 34, 35]. Whether this novel purification strategy might be applied to other disulfide-rich proteins of non-malarial origin remains to be investigated.

In conclusion, we have demonstrated that *L. lactis* is an efficient host for production of *P. falciparum* antigens. We have specifically expanded the toolbox to include a novel procedure based on fusion with IDP-carrier protein and successful heat-based purification for production of disulfide-rich proteins. The efficiency of this technique suggests that it could replace the capturing step by IMAC thus, simplifying and accelerating the purification of recombinant proteins. This approach could also facilitate production of affinity-tag free functional recombinant proteins at industrial-scale for clinical applications.

## Methods

### Preparation of constructs

Target DNA sequences from selected *P. falciparum* genes (Table 1) were amplified by PCR using gene specific primers. For *Sera5*^3D7^ and *MSP2*^FC27^ we used respective synthetic genes (GeneArt^®^ Life Technologies, Germany). All constructs without the GLURP-R0 carrier were cloned into the *Bgl*II restriction site of pSS1 [22]. GLURP fusion proteins were constructed by cloning the target gene into the *Bgl*II restriction site of pSS2 [22]. The target gene was amplified with a forward primer containing the DNA sequence encoding for a TEV protease cleavage site (ENLYFQG) thereby creating a protein fusion with the TEV site in the fusion junction. All constructs were verified by sequencing and subsequently transformed into *Lactococcus lactis* MG1363 by electroporation as described [22].

### Screening and fermentation

Five-ten colonies from each transformation were grown overnight at 30 °C in 5 ml LAB medium supplemented with 4% glycerol–phosphate, 5% glucose and 5 μg/ml erythromycin. Culture supernatants were clarified by centrifugation at 9000*g* for 20 min and protein expression levels were assessed in the culture supernatants by ELISA using HRP-conjugated anti-His antibody (MACS, Miltenyi biotech, Germany) and by SDS-PAGE. *L. lactis* MG1363 harboring expression constructs was grown in a 1 l stirred bioreactors for 15 h at 30 °C [23]. Cells were removed by centrifugation at 9000 rpm for 30 min and the raw culture supernatant was clarified by filtration. Culture-filtrates were either used immediately or stored at − 20 °C.

### Purification of proteins expressed without R0-fusion partner

Culture-filtrates were concentrated fivefold and diafiltrated in quick stand system (GE Healthcare, Sweden) against Tris-buffered saline (TBS) pH 8.0 supplemented with 10 mM imidazole. Recombinant protein was captured from the clarified culture supernatant on a 5 ml HisTrap HP column (GE Healthcare, Sweden). Bound protein was eluted with TBS plus 500 mM imidazole at a flow rate of 4 ml/min and fractions containing the desired protein were pooled and diluted tenfold in 50 mM Tris buffer before loading on the 5 ml HiTrap Q HP or the 5 ml HiTrap SP HP columns (GE Healthcare, Sweden). Bound protein was eluted through gradient elution in 50 mM Tris containing 0–1 M NaCl. Fractions containing the recombinant protein were pooled and buffer exchanged against 50 mM Tris, 300 mM NaCl and 1 mM EDTA, pH 8.0.

### Purification of recombinant proteins expressed with R0-fusion partner

Recombinant fusion proteins were expressed and affinity purified as described above. The purified fusion protein was digested for 16 h at room temperature with the TEV protease [36], diluted tenfold with 50 mM Tris buffer and loaded on to the ion-exchange chromatography column (Table 1). Bound protein was eluted gradient elution in 50 mM Tris containing 0–1 M NaCl thereby separating the target protein from the GLURP fusion partner and the TEV protease. Purified recombinant protein was concentrated by a VIVA spin column 10 kDa cutoffs (Sartorius, UK), and stored in TBS plus 1 mM EDTA at − 80 °C until use. Protein concentration was determined by the BCA protein assay (Thermo Fisher Scientific). Purity of target recombinant proteins were monitored using 4–12% Tris-tricine SDS-PAGE gels. Western blotting was performed using HRP-conjugated anti-His and anti-GLURP-R0 antibodies.

### Protein purification by boiling

Clarified culture-filtrates were concentrated fivefold and incubated in a boiling water-bath for 10 min followed by immediate cooling in an ice-bath. Host cell proteins (HCP) were removed by centrifugation at 30,000 rpm for 15 min at 4 °C and the supernatant containing the GLURP-R0 fusion protein was buffer exchanged against TBS and incubated with the TEV protease for 16 h at room temperature and the target protein was purified as described above.

### SE-HPLC and free thiol determination

Analytical size exclusion high-performance liquid chromatography (SE-HPLC) of purified proteins was performed using an Agilent 1100 Series HPLC System (Agilent Technologies, USA) equipped with a Agilent advance Bio SEC 300 Å, 2.7 μm, 4.6 × 300 mm SEC column, (Agilent Technologies, GB) as per the manufacturer’s instructions. Briefly, five hundred pmol of protein was loaded on the SEC column and eluted with a 0.350 ml/min flow of elution buffer (phosphate buffer) at room temperature. Standard proteins from Sigma Aldrich were also run using same the conditions mentioned above for sizing of the purified recombinant proteins. The absorbance was measured at 280 nm and chromatographic peaks were integrated by HPLC ChemStation (Agilent Technologies, CA, US). The amount of free cysteine residues was measured using Ellman’s Reagent (Thermo fisher scientific, USA) following the manufacturer’s instructions. A standard curve was constructed using known concentrations of free cysteine (Sigma-Aldrich, USA).

### Enzyme linked immunosorbent assay (ELISA) and Multiplex analysis

ELISA was performed as described elsewhere [37, 38]. The coating concentration of recombinant protein was 1 μg/ml. Serum samples were used from hyperimmune adults (malaria infected though clinically healthy male blood donors) living in an area in Liberia where malaria is holoendemic, or from Danish donors never exposed to malaria infection [39]. Antigenicity of the purified recombinant proteins was assessed in a multiplex Luminex assay as described elsewhere [40] with minor modifications. Briefly, approximately 1250 microspheres from each of the 26 antigens coupled bead regions were mixed in three separate mixtures. Plex 1 contained: GLURP-R0, GLURP-R2, cMSP3^3D7^, nMSP3^K1^, MSP3.7, MSP6, Pf38, MSP1_19k_, and BSA; Plex 2 contained, MSPDBL2, SERA5, MSP2^3D7^, Pf41, Pf12, MSP3.3, nMSP3^3D7^ and BSA; and Plex 3 contained MSPDBL1, MSP2^FC27^, GAMA, RAMA, MSPDBL1_Leu_, PfRH2-2030, PfRH2a, PfRH2b, RALP-1, RON4, EBA140RIII-V and BSA. EBA140 RII, RIPR, SUB2, RON2 and PfRh4.2 could not be included in the MPA due to either low protein production or unavailability of recombinant proteins during plex preparation. 50 μl of each Plex mixture was added to a 96-well filter micro titer plate (MSBVS 1210, Millipore). For positive and negative controls, a pool of plasma from five hyperimmune Liberian donors and a pool of plasma from eight Danish donors tested at 1:2000 dilutions were used. Fifty-four hyperimmune Liberian and 100 Danish sera samples are tested in singlet at 1:1000 dilution by adding 100 μl/well and incubated with shaking for 2 h in the dark. After three washes, bound IgG was detected with PE-labeled goat anti-human IgG (Jackson Immuno Research Laboratories, West Grove, PA). Levels of specific antibodies against each antigens were nearly identical either determined by the multiplex assay (all beads in each plex) or by single-antigen assay (single bead/antigen), demonstrating that none of the antigens competed for or bound cross-reactive IgG antibodies (data not shown).

### Disorder prediction

IUPred software was used for the prediction of protein disorder [41]. For analysis of protein disorder, we estimated a disorder score for each recombinant protein by calculating the percentage of residues with a disorder score above 0.7.

### Statistical analysis

The Mann–Whitney rank sum test was used for analyzing differences between groups. P < 0.05 was considered to be statistically significant.

## Additional files

**Additional file 1.** Success rate of obtaining expression of target recombinant protein in *L. lactis* is not dependent on its biophysical characteristics. Success rate of expression of different target recombinant proteins in *L. lactis* have been grouped into High, Medium or Low depending on the yield of the respective expression. Yields show poor co-relations with the protein disorder score and presence of cysteine residues (**a**) and iso-electric point and the predicted molecular weight of the target recombinant proteins (**b**).

**Additional file 2.** Successful expression of different *P. falciparum* antigen derived recombinant proteins in *L. lactis*. Purity profile of different target recombinant proteins as determined by SDS-PAGE analysis as shown by Coomassie blue staining (top panel) or Western Blot analysis (bottom panel) observed with anti-His antibody.

**Additional file 3.** Anomalous migration by SDS-PAGE is related to protein disorder. Anomalous mobility was determined as the ratio between the apparent molecular weight as determined by SDS-PAGE and the molecular weight calculated from the deduced amino acid sequence (Table 1). Protein disorder was predicted using the IUPred software [41]. The protein disorder score was estimated by calculating the percentage of residues with a disorder score above 0.7.

## Abbreviations

DTT: dithiothreitol
SEC: analytical size-exclusion chromatography
ELISA: enzyme linked immunosorbent assay
IEC: ion exchange chromatography
IMAC: immobilized metal ion chromatography
GLURP: glutamate-rich protein of *Plasmodium falciparum*
MSP3: merozoite surface protein 3 of *Plasmodium falciparum*
MSP1_19k_: merozoite surface protein 1 19 kDa
MSP2^3D7^: merozoite surface protein 2 3D7 allelic family
MSP2^FC27^: merozoite surface protein 2 FC27 allelic family
Pf12: merozoite surface protein P12
Pf38: merozoite surface protein P38
nMSP3^3D7^: merozoite surface protein 3 n-terminal 3D7 allelic family
cMSP3^3D7^: merozoite surface protein 3 c-terminal 3D7 allelic family
nMSP3^K1^: merozoite surface protein 3 n-terminal K1 allelic family
MSP6: merozoite surface protein 6
MSP3.3: merozoite surface protein 3.3
MSP3.7: merozoite surface protein 3.7
GLURP-R0: glutamate-rich protein R0
GLURP-R2: glutamate-rich protein R2
SERA5: Serine Repeat Antigen 5
Pf41: merozoite surface protein 41
MSPDBL1: Duffy binding-like merozoite surface protein 1
MSPDBL2: Duffy binding-like merozoite surface protein 2
MSPDBL1_Leu_: Duffy binding-like merozoite surface protein 1 Leucine zipper
EBA140RIII–V: erythrocyte binding antigen region III–V
EBA140 RII: erythrocyte binding antigen region III
GAMA: GPI anchored micronemal antigen
RIPR: reticulocyte binding protein homologue 5-interacting protein
SUB2: subtilisin-like protease 2
PfRh2b: *Plasmodium falciparum* reticulocyte-binding homolog 2b
PfRh2a: *Plasmodium falciparum* reticulocyte-binding homolog 2a
PfRh4.2: *Plasmodium falciparum* reticulocyte-binding homolog 4.2
