# Supplementary figures and images for: Lactococcus lactis provides an efficient platform for production of disulfide-rich recombinant proteins from Plasmodium falciparum
Source: Microb Cell Fact. 2018 Apr 5;17:55. doi: 10.1186/s12934-018-0902-2 (PMC5885415; doi:10.1186/s12934-018-0902-2)

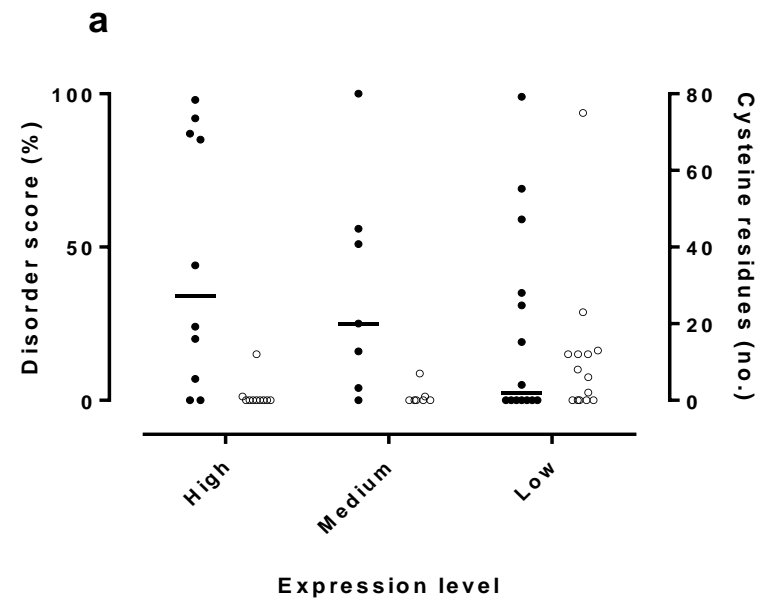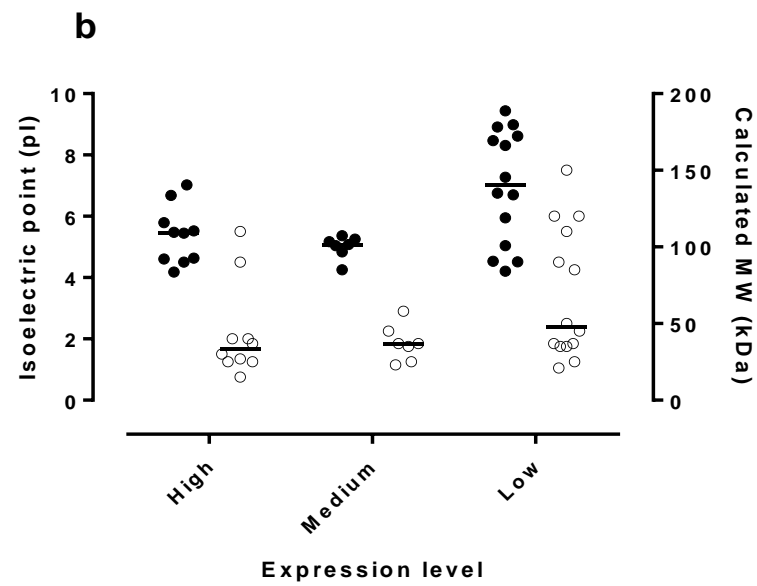

Supplement: Supplementary file 1 — Additional file 1. Success rate of obtaining expression of target recombinant protein in L. lactis is not dependent on its biophysical characteristics. Success rate of expression of different target recombinant proteins in L. lactis have been grouped into High, Medium or Low depending on the yield of the respective expression. Yields show poor co-relations with the protein disorder score and presence of cysteine residues (a) and iso-electric point and the predicted molecular weight of the target recombinant proteins (b). [file 12934_2018_902_MOESM1_ESM.pdf]

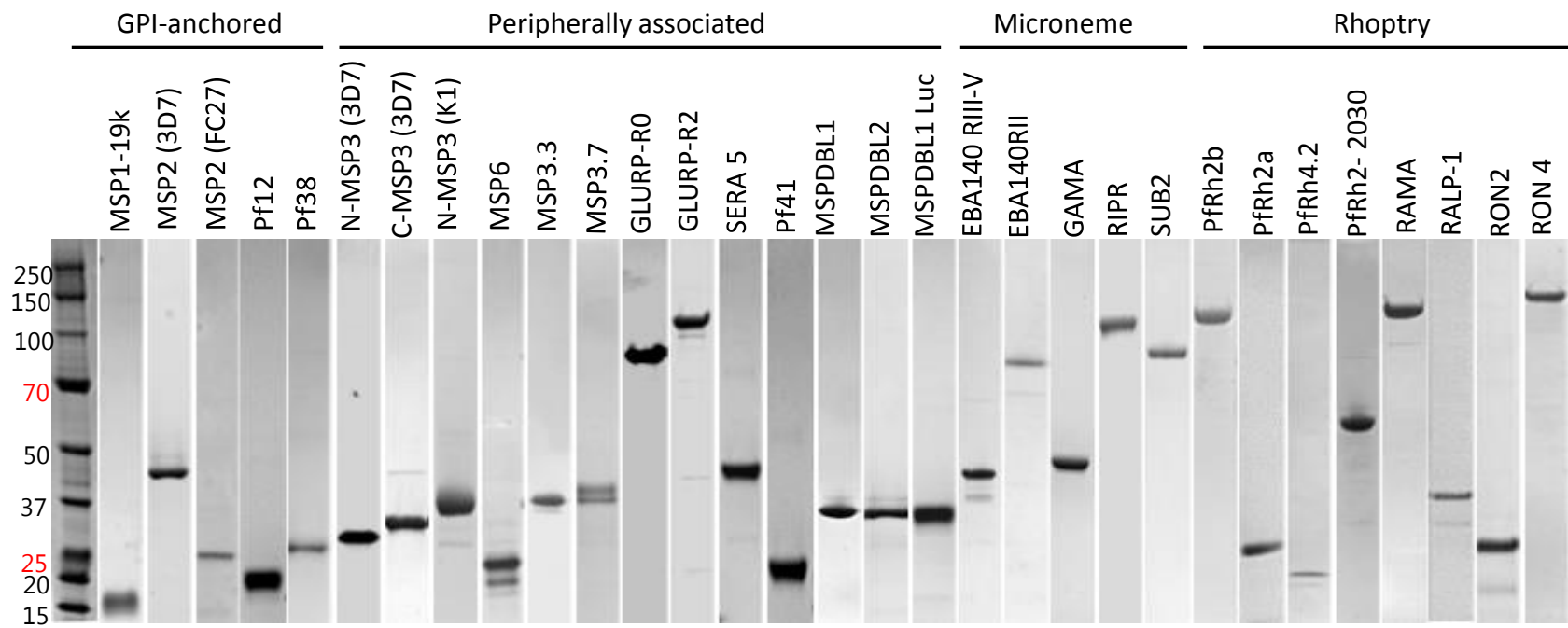

Coomassie

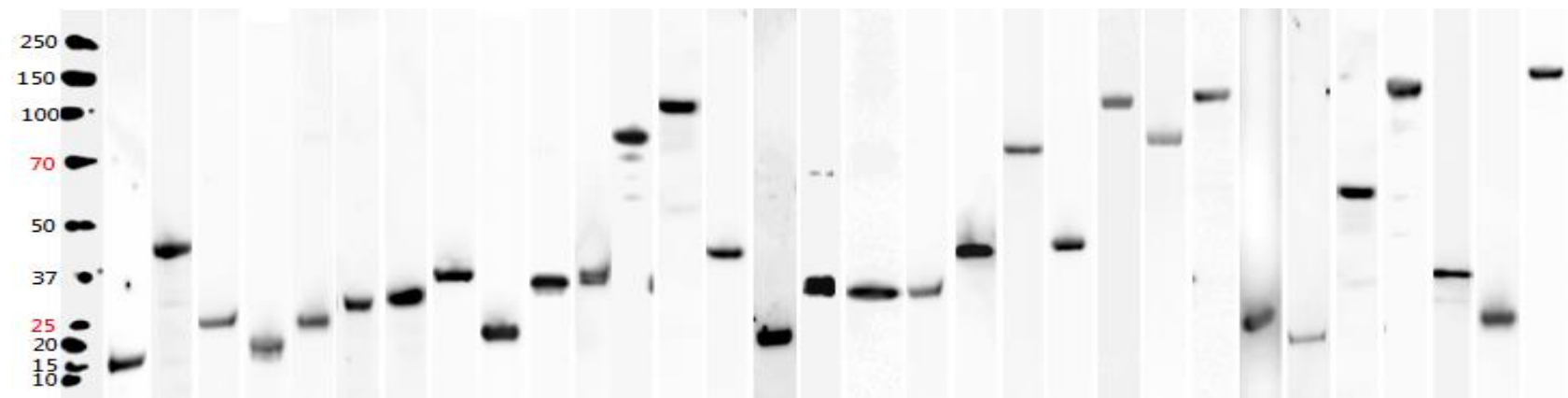

Anti-His

Supplement: Supplementary file 2 — Additional file 2. Successful expression of different P. falciparum antigen derived recombinant proteins in L. lactis. Purity profile of different target recombinant proteins as determined by SDS-PAGE analysis as shown by Coomassie blue staining (top panel) or Western Blot analysis (bottom panel) observed with anti-His antibody. [file 12934_2018_902_MOESM2_ESM.pdf]

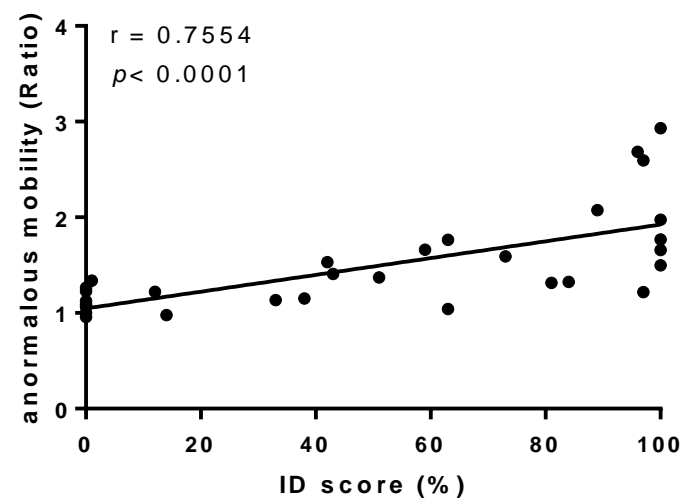

Supplement: Supplementary file 3 — Additional file 3. Anomalous migration by SDS-PAGE is related to protein disorder. Anomalous mobility was determined as the ratio between the apparent molecular weight as determined by SDS-PAGE and the molecular weight calculated from the deduced amino acid sequence (Table 1). Protein disorder was predicted using the IUPred software [41]. The protein disorder score was estimated by calculating the percentage of residues with a disorder score above 0.7. [file 12934_2018_902_MOESM3_ESM.pdf]
